# A Rare Case of Burkitt’s Lymphoma Presenting With Features Mimicking Vogt-Koyanagi-Harada Disease

**DOI:** 10.7759/cureus.17659

**Published:** 2021-09-02

**Authors:** Ramya Tadipatri, Daniel Gonzalez, Suraj Muley, Ekokobe Fonkem, Amir Azadi

**Affiliations:** 1 Neurology, St. Joseph's Hospital and Medical Center, Phoenix, USA

**Keywords:** burkitt's lymphoma, hiv, aids, vogt koyanagi harada disease, uveitis, retinal detachment

## Abstract

Here, we report a case of Burkitt’s lymphoma in an HIV-positive patient presenting with features suggestive of Vogt-Koyanagi-Harada disease (VKHD), which in retrospect was likely a misdiagnosis. We hope to describe a rare presentation of lymphoma in order to prevent misdiagnosis and promote early recognition. The patient was a 25-year-old male who initially presented with right eye pain and blurry vision. He was found to have bilateral serous retinal detachments and was diagnosed with VKHD and started on prednisone. He stopped taking the prednisone, and his vision worsened. He then developed right eye ptosis, restricted eye movements, nausea, vomiting, headache, dysphagia, tongue deviation, and slurred speech. MRI showed diffuse cranial nerve enhancement. He was found to be positive for HIV and Hepatitis A with CD4 count of 41. Lumbar puncture showed WBC 83 (94% lymphocytes), RBC 1460, glucose 62, and protein 195, with Epstein-Barr virus (EBV) positivity and negative cytology. Gd1a antibody was positive (72). He underwent empiric treatment with IV solumedrol for possible VKHD exacerbation, followed by empiric intravenous immune globulin (IVIG) for possible acute inflammatory demyelinating polyneuropathy (AIDP). He subsequently developed diffuse limb weakness and loss of reflexes, and he was treated with plasma exchange (PLEX). He demonstrated minimal response to treatment. Electromyography (EMG) was unrevealing, and the MRI of the cervical and lumbar spine showed diffuse nerve root thickening and enhancement. He underwent an esophagogastroduodenoscopy (EGD) for continued dysphagia, and the biopsy was positive for an aggressive B-cell lymphoma strongly favoring Burkitt’s lymphoma. VKHD is a rare condition diagnosed based on retinal exam findings. Few cases of lymphoma report findings suggestive of VKHD. This is a rare case of lymphoma initially presenting with these retinal findings. Understanding this potential presentation of lymphoma is essential for early diagnosis and treatment and for optimizing patient outcomes.

## Introduction

Vogt-Koyanagi-Harada disease (VKHD) is a systemic granulomatous autoimmune disease process that preferentially invades tissues rich in melanocytes and is characterized by panuveitis, often with associated neurological and cutaneous manifestations. It is predominantly seen in populations with pigmented skin including Asians, Middle Easterners, Hispanics, and Native Americans, although it is very infrequent among people of African descent [1].

VKHD is divided into four stages: prodromal, acute uveitic, convalescent, and chronic/recurrent [1]. The prodromal stage lasts a few days and presents with fever, headache, nausea, and orbital pain. Cerebrospinal fluid (CSF) pleocytosis can be observed [1]. The acute uveitic stage occurs within three to five days of the prodromal stage and may be present for weeks. Patients report blurring of vision in both eyes due to diffuse choroiditis. Ophthalmologic findings include exudative detachment of the retina, hyperemia and edema of the optic disk, and multiple hyperfluorescent leaking dots referred to as “pinpoints” on fluorescein angiography [1]. The convalescent stage occurs a few months after the acute uveitic stage and is characterized by depigmentation of the integument and choroid, vitiligo, alopecia, and poliosis, with fundus appearing an exaggerated red, known as “sunset glow fundus” [1]. The recurrent or chronic stage occurs within the first three to six months of diagnosis, typically following taper of corticosteroids, and involves cataract formation, glaucoma, choroidal neovascularization, and retinal choroidal fibrosis [1].

The pathophysiology of VKHD is primarily driven by a T-cell-mediated immune process against melanocytes that express class II MHC. Cytokines produced by Th1 helper cells, specifically IL-2 and interferon-gamma, may have a role in the granulomatous choroidal inflammation in the acute phase of VKHD [1]. The diminished regulatory T-cell function has also been described [1]. Given that it is a T-cell mediated response, VKHD is extremely rare among HIV patients, with only very few cases reported in the literature [2].

The differential diagnosis for VKHD includes primary intraocular lymphoma, which is a subtype of non-Hodgkin central nervous system lymphoma. Ophthalmologic findings can be similar, with multifocal subretinal and sub-RPE yellow lesions involving the posterior pole associated with vitritis, satellite lesions simulating hypopigmented lesions in the mid-periphery, choroidal thickening with or without retinal detachment, and choroidal fluorescence blockage with late leakage at the site of inflammatory lesions on fluorescein angiography. However, unlike VKHD, later phases of the angiogram reveal extensive focal early hyperfluorescence with dye pooling in the detached retina region [1]. Additionally, there have been a few cases in the literature that have highlighted a possible association between VKHD and lymphoma.

Among HIV patients, the risk of non-Hodgkin’s lymphoma is increased from 60- to 200-fold compared to the general population. This has been attributed to multiple factors including immunosuppression and cytokine dysregulation, opportunistic infection with EBV and HHV8, and the transforming properties of the retrovirus itself. EBV in particular is thought to play a prominent role, given its presence in 40%-50% of HIV-associated lymphomas. Burkitt’s lymphoma is the most common subtype, comprising 35%-50% of HIV-associated non-Hodgkin’s lymphoma cases. The diagnosis requires a medium-sized CD10-positive B-cell population with a high proliferation rate as well as MYC gene translocation. EBV positivity ranges from 30% to 70% depending on subtype. Clinical outcomes are worse among HIV patients, with 80% presenting with advanced systemic disease. Following the advent of highly active antiretroviral therapy (HAART), the incidence of lymphoma in HIV patients has been drastically reduced [3]. The CHOP (cyclophosphamide, doxorubicin, vincristine, prednisone) regimen, commonly used for diffuse large B-cell lymphomas, has not been shown to be effective in Burkitt’s lymphoma, producing high recurrence rates. Chemotherapy regimens used for the treatment of Burkitt’s lymphoma include the CODOX-M/IVAC (cyclophosphamide, doxorubicin, vincristine, methotrexate, ifosfamide, cytarabine, etoposide) regimen and DA-REPOCH (etoposide, prednisone, vincristine, cyclophosphamide, doxorubicin, rituximab) regimen. The CODOX-M/IVAC regimen was found to be more effective in younger patients. Patients experienced similar outcomes regardless of HIV status [4-6].

Here, we describe a case of Burkitt’s lymphoma presenting with findings that appeared to mimic VKHD. This type of mimicry has previously only been clearly described rarely in literature, and lymphoma presenting initially with findings suggestive of VKHD has not been previously described.

An abstract of this report was previously presented at the 2020 AAN Science Highlights Virtual Poster Session.

## Case presentation

The patient is a 25-year-old African American male who initially presented with right eye pain and bilateral blurry vision. A month later, he was seen by a retinal specialist and underwent fluorescein angiography that revealed bilateral serous retinal detachments. He was diagnosed with VKHD and was started on prednisone 60 mg daily, with a plan to taper by 10 mg over the course of four weeks.

He was initially compliant with his steroid regimen, but due to symptoms of anxiety and jitteriness, he stopped taking it after three weeks. Subsequently, his vision worsened, and a few days later, he developed right eye ptosis, nausea, vomiting, headache, and difficulty swallowing liquids.

He presented to the emergency room a few days after. Examination revealed right eye ptosis with a 7-mm unreactive pupil and limited movement in all directions as well as impaired abduction of the left eye. He underwent MRI brain, MRA head and neck, and MR orbits, although only non-contrast images were obtained due to patient anxiety and body habitus, and all imaging sequences were unremarkable. Prednisone was recommenced 60 mg daily.

**Figure 1:**
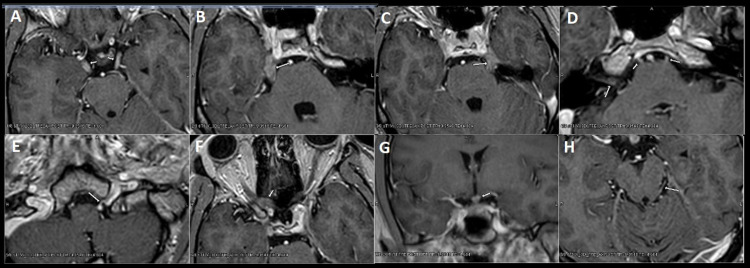
MRI brain images demonstrating multiple cranial nerve thickening and enhancement Axial post-contrast MRI sequences depicting thickening and enhancement of the (A) bilateral optic tracts, (B) right trigeminal nerve, (C) left trigeminal nerve, (D) bilateral abducens nerves and right facial nerve, (E) left hypoglossal nerve, (F) right optic nerve, (G) infundibulum, and (H) left trochlear nerve.

About two days later, he was run as a stroke code with emergent imaging for new-onset tongue deviation, slurred speech, and diminished hearing in his right ear. MRI brain with and without contrast noted extensive cranial nerve thickening and enhancement, including bilateral cranial nerves II and III, left cranial nerve IV, bilateral cranial nerve V, left cranial nerve VI, right greater than left cranial nerve VII and VIII, and left cranial nerve XII (Figures 1A-1H). Differential diagnosis at this time included progression of VKHD, infectious or inflammatory granulomatous processes, neoplastic involvement, or an idiopathic inflammatory polyneuropathy with cranial nerve involvement such as Miller Fisher variant versus a hereditary polyneuropathy or neurofibromatosis.

He was newly diagnosed with HIV based on positive HIV1 antibody titer, with 63,000 copies/mL and CD4 count 41 cells/μL. Further infectious workup returned positive for Hepatitis A, with negative antibodies for CMV, HSV, VZV, Hepatitis B, toxoplasma, coccidioidomycosis, aspergillus, gonorrhea, and chlamydia, as well as negative RPR and quantiferon. Lumbar puncture was performed and revealed glucose 62, protein 195, WBC 83 with 94% lymphocytes, and RBC 1460. CSF was negative for oligoclonal bands, with normal MBP and IgG index. CSF cytology was negative, and flow cytometry was not obtained. CSF infectious workup was positive for EBV with 150,000 copies/mL and was negative for HSV 1/2, cytomegalovirus (CMV), varicella zoster virus (VZV), HHV 6/7, mumps, measles, West Nile virus, mycobacterium, enterovirus, and cryptococcus. Serum and cerebrospinal fluid (CSF) bacterial, fungal, and AFB cultures were negative. Serum autoimmune panel returned positive for ANA with mildly elevated RNP of 1.1 and elevated SS-A of 2.4. Ganglioside panel was notable only for elevated GD1a antibody of 72. Serum and CSF paraneoplastic panel including LGI1, CASPR2, AMPAR 1/2, GABAR, and NMDAR were negative.

He was started on sulfamethoxazole/trimethoprim, fluconazole, and azithromycin prophylaxis for his low CD4 count. Given his symptomatic worsening following cessation of steroids, he was treated empirically for possible VKHD exacerbation with IV methylprednisolone 1000 mg daily for three days and was continued on prednisone 60 mg daily. This was followed by empiric treatment for possible acute inflammatory demyelinating polyneuropathy (AIDP) with IVIG 0.5 g/kg divided over four days. On day 9 of hospitalization following completion of IVIG, he was started on emtricitabine and raltegravir. His right eye ptosis improved, but on day 10, he started to develop a left eye third nerve palsy with restricted eye movements. On day 11, he was noted to have a loss of reflexes. On day 13, he started to complain of dysphagia and dysarthria and developed limb weakness (4-/5 ankle dorsiflexion, 4-/5 hip flexion, 4-/5 finger abduction, 4+/5 elbow extension, and 4/5 shoulder abduction), so he was treated with a five-day course of plasma exchange (PLEX) for presumed AIDP. On day 14, a fiberoptic endoscopic evaluation of swallowing was performed noting right vocal cord paralysis, edema, and copious secretions. On day 16, EMG of the left upper and lower extremity was performed, which revealed only a left ulnar motor neuropathy, and MRI of the cervical and lumbar spine showed thickening and enhancement of multiple ventral and dorsal cervical and lumbar nerve roots. Due to continued dysphagia, he underwent EGD with gastric and duodenal biopsies and percutaneous endoscopic gastrostomy (PEG) placement. He had nominal subjective improvement in strength and return of biceps reflex (1+), therefore was discharged to a rehabilitation center on prednisone 60 mg daily.

One week later, he was readmitted due to persistent nausea and vomiting. CT of chest, abdomen, and pelvis areas was negative. Duodenal and gastric biopsy from prior admission revealed an atypical lamina propria lymphoid infiltrate favoring aggressive B-cell lymphoma with MYC rearrangement, scattered cells positive for CMV. Further characterization of the lymphoma cells demonstrated MIB1 approaching 100%, intermediate size, prominent nucleoli, variable nuclear contours, and cytoplasmic vacuolization. MUM-1 was negative as were CD30, CD43, CD5, CD3, and BCL-2. Stains for PAX5, CD79a, BCL-6, CD10, and CD20 were positive. Fluorescence in situ hybridization (FISH) panel showed an MYC gene rearrangement without BCL-2 or BCL-6 abnormalities. Burkitt’s lymphoma was strongly favored, with differential also including large B-cell lymphoma or high-grade B-cell lymphoma not otherwise specified. A bone marrow biopsy was performed and found negative for malignancy. He was started on CODOX-M/IVAC chemotherapy treatment. His course was complicated by febrile neutropenia, septic shock, and appendicitis requiring appendectomy, and a decision was made to withdraw care and transfer to hospice.

## Discussion

Although the patient had presented to us having received a diagnosis of VKHD, this appears to have been a misdiagnosis. His ophthalmological findings were instead likely to have been a presenting sign of his underlying lymphoma. There have been a few cases reported in the literature describing concurrent diagnoses of VKHD and lymphoma, with some skepticism raised regarding the accuracy of the VKHD diagnosis.

Hashida et al. described a case of a 69-year-old male with prior history of gastric diffuse intermediate B-cell large-type malignant lymphoma in remission following chemotherapy and total gastrectomy, presenting with sudden bilateral loss of visual acuity [7]. He was found to have moderate anterior chamber cells with granulomatous keratic precipitates on the retrocornea in both eyes and a circumferential posterior synechia in the left eye as well as bilateral papilledema and serous retinal detachment. He had elevated levels of soluble IL-2 receptor (sIL-2R) (1954 U/mL), which has been identified as a tumor marker for malignant lymphoma. HIV status was not reported. He was treated with bone marrow radiation and CHOP. He had improvement in his visual acuity over the course of treatment but eventually developed graying of hair, leukoderma on the forehead and upper limbs, degeneration of retinal pigment epithelium, and bilateral sunset glow fundus. The elevated sIL-2R levels were attributed to malignant lymphoma as sIL-2R has not been shown to be elevated in VKHD [8]. Given that VKHD is a Th1-mediated phenomenon, the authors postulated that high sIL-2R that results in a predominance of Th1-type helper T cells may have induced the melanocyte-specific T-cell response classically observed in VKHD.

Pahk et al. described a 56-year-old male presenting with fever of unknown origin, followed by decreased visual acuity bilaterally four months later [9]. He was noted to have multiple bilateral retinal detachments involving the macula, with fluorescein angiography further demonstrating choroidal hypofluorescence and spotted choroidal hyperfluorescence suggestive of choroiditis as well as patches of early perivascular hyperfluorescence with leakage. HIV was nonreactive. He was diagnosed with VKHD and treated with prednisone, resulting in complete resolution of serous detachment, although perivascular staining was still present. He was later diagnosed with intravascular lymphoma based on skin biopsy. He was treated with CHOP-R (cyclophosphamide-hydroxydaunorubicin-Oncovin-prednisone-rituximab) leading to resolution of perivascular staining. The authors postulated that the perivascular hyperfluorescence had in fact represented clusters of tumor cells in the retinal vasculature.

Elevated sIL-2R is observed in certain malignancies including non-Hodgkin’s lymphoma (NHL). In particular, levels greater than 2000 U/mL correlate with high tumor burden at diagnosis, B symptoms, and poor response to therapy. As a result, it has been proposed as a biomarker for prognosis of NHL patients [10]. Furthermore, sIL-2R has been found to increase as a direct result of HIV infection. Hofmann et al. found that sIL-2R increased over baseline in 83% of male HIV patients within six months of seroconversion. They also found that a sample of HIV seropositive men had increased sIL-2R levels compared to seronegative men, which were statistically significant and double the median value [11]. Although sIL-2R was not measured in our patient, it may have potential use as both a screening and prognostic biomarker.

Of note, our patient had a substantially lower age at presentation than the patients described by Hashida et al. and Pahk et al. Age-related molecular heterogeneity has been described in both sporadic and AIDS-related Burkitt’s lymphoma, and this has translated to differences in survival and response to treatment [12]. Taken together, it appears that the ocular findings described can be seen in lymphoma patients regardless of age, subtype, or HIV status.

## Conclusions

Based on our patient’s HIV status, EBV positivity, MYC rearrangement on biopsy, and highly suggestive histology including CD10 and CD20 positivity with high proliferative rate, his likely diagnosis was Burkitt’s lymphoma with an unclear primary source. Thus, future misdiagnosis of Burkitt’s lymphoma could be prevented if the ocular findings observed in our patient were more widely recognized as a potential presentation of the disease. This was unlikely to have been a primary intraocular lymphoma, given the patient’s age and low prevalence of the condition among HIV patients. Diffuse enhancement of multiple cranial and peripheral nerve roots was ultimately attributed to neurolymphomatosis.

Given the proposed link between VKHD and lymphoma as mentioned above, based on sIL-2R levels, there may be a common pathophysiologic mechanism underlying the masquerade phenomenon that we have observed. This supports the idea that lymphoma workup may be warranted in newly diagnosed VKHD, with consideration for retinal or choroidal biopsy, particularly if other risk factors such as HIV reactivity are present.
